# The usefulness of a newly developed ultrasonographic measurement method for predicting post-operative stiffness after arthroscopic rotator cuff repair

**DOI:** 10.1016/j.jseint.2026.101789

**Published:** 2026-09-04

**Authors:** Yuki Miyasaka, Takahiro Sekiguchi, Norimasa Takahashi, Toshiki Nagaoka, Keisuke Kimura, Kohei Matsuda, Keisuke Matsuki, Shota Hoshika, Hiroyuki Sugaya, Itaru Kawashima

**Affiliations:** a Department of Rehabilitation, Funabashi Orthopaedic Clinic, Funabashi, Chiba, Japan; b Sports Medicine and Joint Center, Funabashi Orthopaedic Hospital, Funabashi, Chiba, Japan; c Medical Corporation Tanaka Clinic, Sendai-shi, Miyagi, Japan; d Takeda Orthopaedic Clinic, Koriyama, Fukushima, Japan; e Tamura Orthopedics Clinic, Shizuoka-si, Shizuoka, Japan; f Tokyo Sports & Orthopaedic Clinic, Toshima-ku, Tokyo, Japan; g Department of Orthopaedic Surgery and Sports Medicine, University of Florida, Gainesville, FL, USA; h Department of Orthopaedic Surgery, Yachiyo Hospital, Anjo, Aichi, Japan

**Keywords:** Rotator cuff tear, Stiffness, Arthroscopic rotator cuff repair, Ultrasonography, Range of motion, Rehabilitation

## Abstract

**Background:**

Post-operative shoulder stiffness (POSS) is one of the most common complications following arthroscopic rotator cuff repair (ARCR) and is thought to be caused by pathologic changes in the anterosuperior soft-tissue structures of the glenohumeral joint. The aim of this study was to determine whether objective ultrasonographic evaluation can predict the occurrence of POSS at 1 year after surgery and to investigate the reliability of a newly developed ultrasonographic measurement method.

**Methods:**

This retrospective cohort study included patients who underwent ARCR at a single institution between April 2017 and June 2018. Ultrasonographic external rotation (UER) and the subscapularis (SSC) sliding rate, representing anterosuperior soft-tissue sliding mobility assessed by ultrasonography, were evaluated at 1 and 3 months post-operatively. POSS was defined as passive external rotation <30° and forward elevation <120° at 12 months. Patients were assigned to 1 of the 2 groups: Group N (without POSS) or Group S (with POSS). Propensity score matching was performed to minimize baseline differences between groups. UER and SSC sliding rate were compared between the groups. Receiver operating characteristic curve analysis was also used to determine predictive thresholds values for POSS at 12 months after surgery, and intrarater and inter-rater reliability were assessed using intraclass correlation coefficients.

**Results:**

After propensity score matching, 40 shoulders (20 per group) were analyzed. The SSC sliding rate at 1 and 3 months and UER at 3 months were significantly greater in Group N than in Group S. The greatest between-group difference was observed for the SSC sliding rate at 1 month post-operatively. Regarding receiver operating characteristic curve analysis, the thresholds for POSS at 1 year post-operatively were 48% for the SSC sliding rate at 1 month after surgery, 38% for the SSC sliding rate at 3 months post-operatively, and 7° for UER at 3 months post-operatively. Reliability analysis showed substantial to almost perfect intrarater and inter-rater reliability for both SSC sliding rate and UER.

**Conclusion:**

Early post-operative ultrasonographic evaluation is useful for predicting the development of POSS at 1 year after ARCR. Reduced SSC sliding rate and limited UER in the early post-operative period were significantly associated with subsequent POSS.

Arthroscopic rotator cuff repair (ARCR) is a standard surgical intervention for rotator cuff tears and generally results in favorable clinical outcomes.^29^ Nevertheless, post-operative shoulder stiffness (POSS)—particularly limited external rotation (ER)—remains a prevalent complication. This condition often leads to persistent discomfort and diminished patient-reported outcomes.^1^^,^^15^^,^^23^ It is widely recognized that pathologic changes in anterosuperior soft-tissue structures, including the subscapularis (SSC) tendon, coracohumeral ligament, subacromial bursa, anterior joint capsule, and supraspinatus muscle, play a role in the development of stiffness.^5^^,^^8^^,^^9^^,^^13^^,^^22^^,^^30^ Consequently, early objective identification of these tissue changes is essential for tailoring physiotherapy to prevent long-term POSS.

Ultrasonography is often used for the diagnosis of rotator cuff tears.^20^^,^^32^ In addition, ultrasonography has been used to assess the sliding mobility of soft tissue such as fascia.^25^^,^^26^ However, these measurement methods have been limited to the thoracolumbar, abdominal and lower limb regions. Methods for evaluating the glenohumeral joint remain largely unexplored.^26^^,^^27^ If measurement techniques for the glenohumeral joint—particularly targeting the soft tissues of the anterosuperior region—were established, they could enable objective assessment of the regions responsible for POSS following ARCR. Although several studies have attempted to predict the occurrence of POSS more than a year after surgery based on relatively early post-operative stages, these reports have lacked objective findings.^19^^,^^31^ Therefore, early prediction of POSS using ultrasonography, particularly through focused evaluation of the anterosuperior region of the glenohumeral joint, is desirable, as this may allow for more objective and reproducible assessment.

We previously reported the clinical significance of early post-operative adduction restriction using the same patient cohort.^17^ Building upon those findings, the present study takes a further step by investigating the predictive value of dynamic soft-tissue sliding mobility—a novel perspective evaluated via ultrasonography—for identifying long-term post-operative stiffness. Specifically, the primary aim of this study was to examine whether objective ultrasonographic evaluation at a relatively early post-operative stage is useful for predicting the occurrence of POSS at 1 year after surgery. The secondary aim was to investigate the reliability of a novel ultrasonographic measurement method. We hypothesized that early ultrasonographic evaluation would be effective in predicting the occurrence of POSS at 1 year post-operatively and that the measurement technique would demonstrate high reliability.

## Materials and methods

### Patients

This retrospective cohort study was approved by the institutional review board and ethics committee of our institution. Patients who underwent ARCR at a single sports medicine center specializing in shoulder surgery between April 2017 and June 2018 and agreed to participate in this study were included. The exclusion criteria were as follows: (1) isolated SSC tendon repair, (2) massive/irreparable rotator cuff tears, (3) a follow-up duration of less than 12 months, and (4) incomplete range of motion (ROM) data. Patient records, including surgical reports, were reviewed for pre-operative demographic information (diagnosis, sex, age at surgery, body mass index [BMI], and operative side) and for rotator cuff tear size, categorized as incomplete tear, small tear (less than 1 cm in length), medium tear (1-3 cm), or large tear (3-5 cm).^6^ We also investigated the presence or absence of SSC tendon tear.

### Surgical procedure

Surgeries were performed with patients in the beach chair position under general anesthesia combined with an interscalene block. A senior shoulder specialist either performed the procedure or supervised a fellow surgeon, ensuring a standardized surgical approach. Following arthroscopic exploration of the glenohumeral joint and subacromial space, subacromial decompression was conducted in every case. If limited tendon mobility was observed, we performed coracohumeral ligament resection and capsular release. Partial-thickness tears were converted to full-thickness and repaired, except when less than 50% of the thickness was involved, in which case only the superficial layer was addressed.

For the repair, triple-loaded suture anchors (HEALIX ADVANCE BR, Johnson & Johnson, New Brunswick, NJ, USA; Depuy Synthes) were placed at the medial margin of the greater tuberosity, and suture limbs were passed through the cuff using a suture grasper. These were secured laterally with knotless anchors (HEALIX ADVANCE Knotless, Johnson & Johnson; Depuy Synthes or SwiveLock C; Arthrex, Naples, FL, USA). Remaining sutures were tied to distribute stress away from the medial row.^29^ The number of anchors varied by tear size, typically using 1–2 for smaller and 2–3 for larger tears. A suture-bridge technique was applied if a concomitant SSC tear was present.^24^ Additionally, the long head of the biceps was managed via tenodesis or tenotomy in cases of medium-to-large tears or evident long head of the biceps pathology.^12^ Our general practice was tenodesis for males under 70 years and females under 65 years.

### Post-operative rehabilitation

A sling with an abduction pillow was used for immobilization for 4 to 5 weeks. On the first post-operative day, patients began isometric cuff training and shoulder girdle relaxation exercises. Passive and active-assisted ROM exercises for flexion and ER were introduced after the sling was discontinued, with strict instructions to avoid provoking pain. Strength training for the rotator cuff and scapular stabilizers commenced at six weeks. Physiotherapy sessions occurred once or twice weekly, supplemented by daily home exercises. Return to light sports was allowed at three months, with a full return to unrestricted activities typically occurring by six months, guided by the patient's functional progress.^11^^,^^16^^,^^24^^,^^25^^,^^29^

### Assessment of range of motion

ROM was assessed at 1, 3, and 12 months post-operatively. Passive forward elevation, abduction, and ER were measured using a hand-held goniometer. Measurement was conducted in a seated position. Active internal rotation was assessed with a tape measure in a standing position, recording the distance (in centimeters) from the spinous process of the seventh cervical vertebra to the thumb.

POSS was defined as passive ER at the side of less than 30° and forward elevation of less than 120° at 12 months after surgery.^4^^,^^21^^,^^34^ Thus, patients were assigned to 1 of the 2 groups: Group N, without POSS or Group S, with POSS.

### Ultrasonographic external rotation evaluation

Ultrasonographic external rotation (UER) was assessed at 1 and 3 months post-operatively. Ultrasonography (FUJIFILM Healthcare Corporation, Tokyo, Japan [Note: formerly Hitachi Aloka Medical]) was used to evaluate the pure ER angle of the glenohumeral joint, referred to as UER. Ultrasonographic images were acquired at a frame rate of 26 fps. The ultrasound operator stabilized the scapula and positioned the probe so that both the SSC tendon and the lower end of the coracoid process could be visualized simultaneously (Fig. 1, *a*–*c*). Next, the patients actively rotated their arm from maximum internal to maximum ER. The active ER movement was executed over 3 seconds. Subsequently, the examiner further externally rotated the glenohumeral joint passively, and experienced physical therapists measured passive ER ROM within a painless range (Fig. 1, *a*). They had previously standardized their measurement methods before the start of the study.

**Figure 1:**
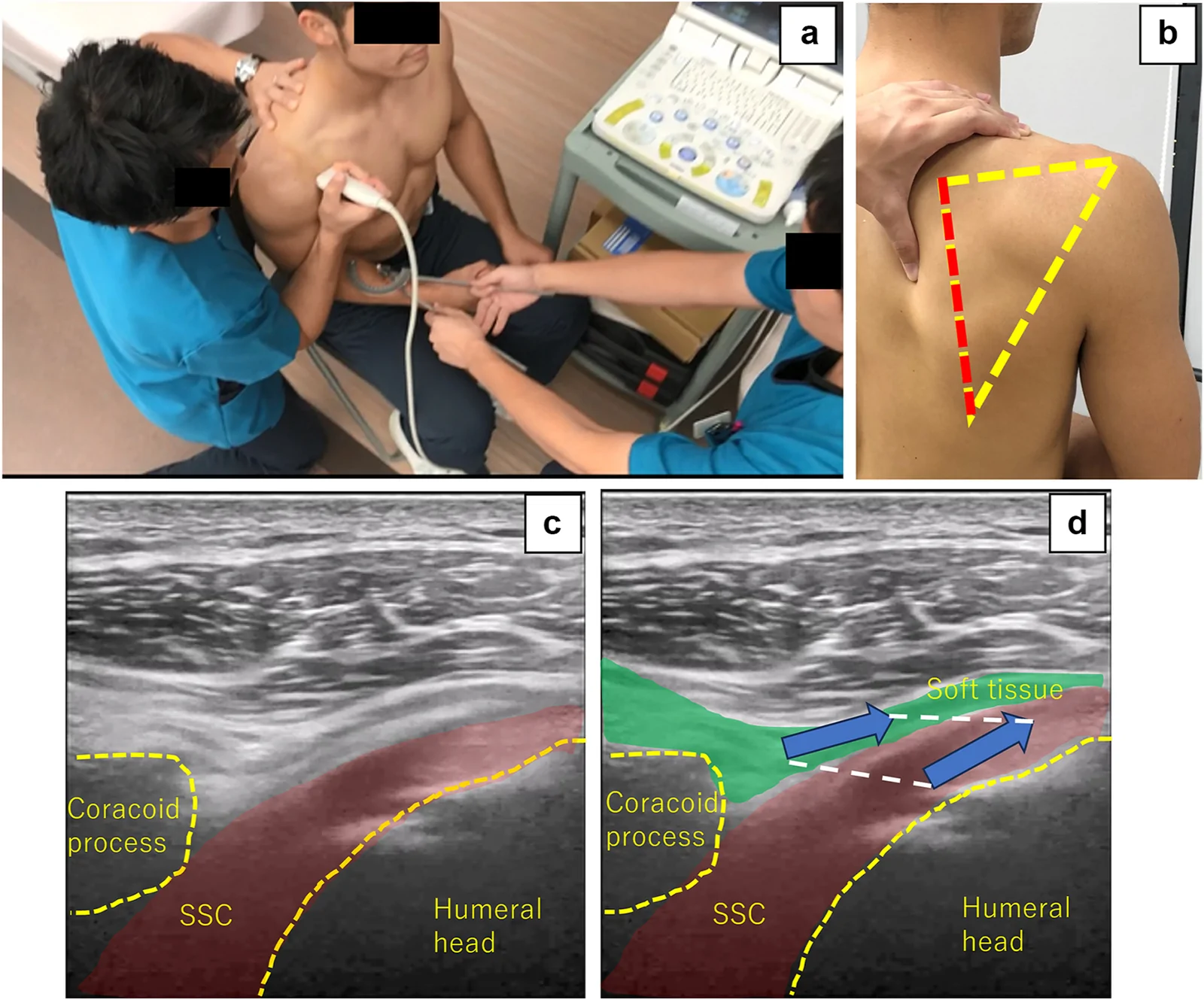
The method for assessing external rotation with ultrasonography and sliding mobility of anterosuperior soft tissue. (**a**) The ultrasound operator fixed the scapula and placed the probe along the long axis of the SSC tendon at the lower end of the coracoid process. The examiner measured active and passive external rotation ROM with ultrasonography (UER) when external rotation was completed. (**b**) The medial border of the scapula was fixed to ensure that the scapula did not tilt anteriorly and downwardly from its starting position. (**c**) The view of ultrasonography. The probe was placed along the long axis of the SSC tendon at the lower end of the coracoid process. (**d**) The formula of SSC sliding rate. The sliding time was divided by the external rotation time and converted to percentages to calculate the SSC sliding rate. *ROM*, range of motion; *SSC*, subscapularis; *UER*, ultrasonographic external rotation.

### Ultrasonographic anterosuperior soft tissue sliding mobility assessment

All measurements were analyzed post hoc using recorded video clips. At first, the time during which ER occurred was identified from the start to the end of the humeral head rotation from the video of the UER (ER time). Next, the time from the start of rotational movement to the point where the sliding between the SSC tendon and the soft tissue directly above it stops sliding was extracted as the sliding time. The cessation of SSC tendon sliding was defined as the point at which the tendon no longer moved independently relative to the overlying superficial soft tissue. This point was determined retrospectively by reviewing the recorded video clips frame by frame. Finally, the sliding time was divided by the ER time and converted to percentages to calculate the SSC sliding rate (Fig. 1, *d*). The SSC sliding rate reflects the degree of tendon mobility, with values closer to 100% indicating better sliding and values closer to 0% indicating poorer sliding (Fig. 2). The SSC sliding rate was assessed at 1 and 3 months post-operatively. All measurements were performed by a physical therapist with more than 10 years of clinical experience. A single trial per shoulder was recorded and analyzed without averaging across multiple repetitions.

**Figure 2:**
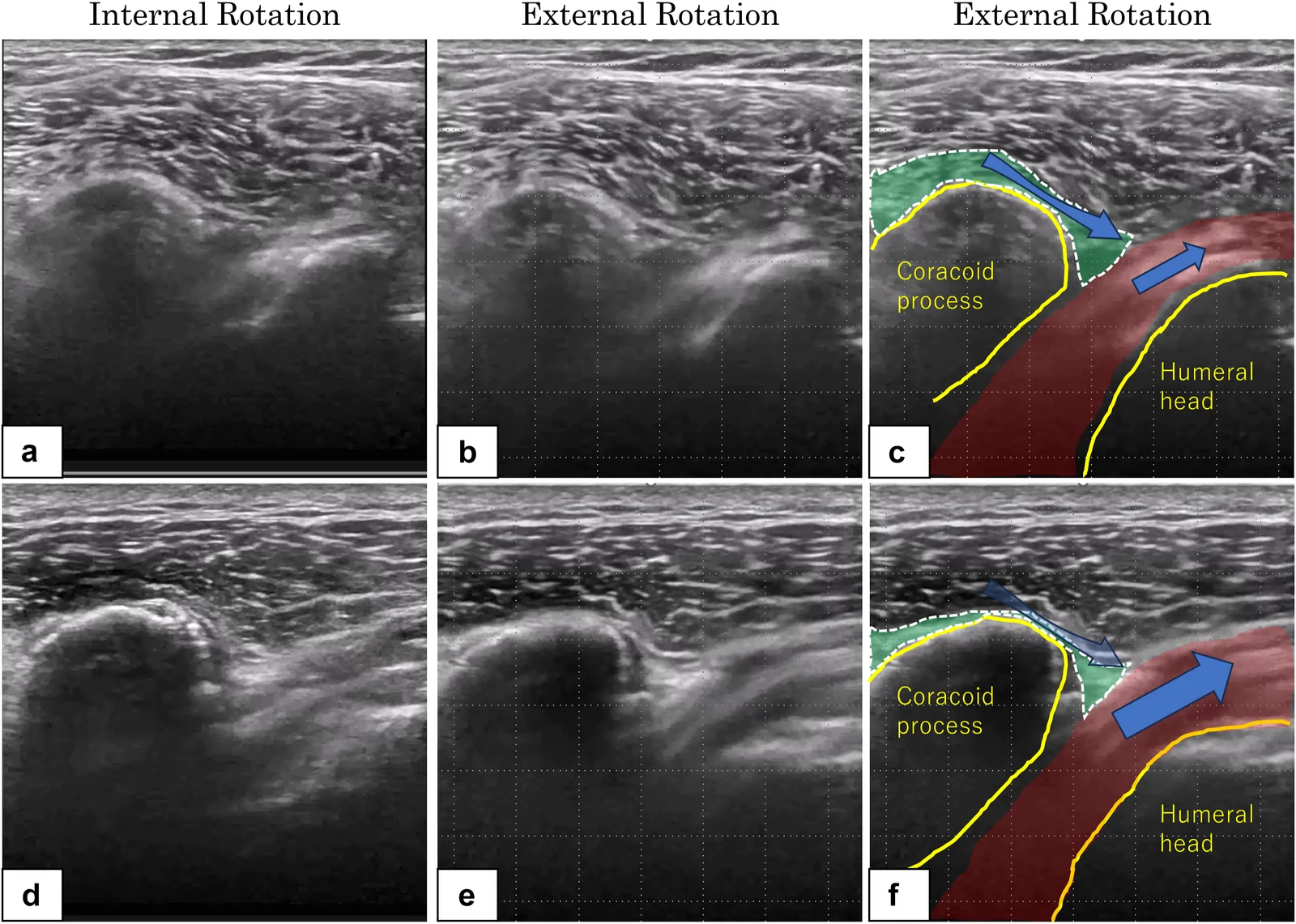
Representative cases of good and poor SSC sliding rates. (**a**), (**b**), and (**c**) Representative case of a poor SSC sliding rate. (**a**) Internal rotation. (**b**) External rotation. (**c**) Sliding condition of the subscapularis tendon. During internal-to-external rotation, the SSC tendon moves together with the adjacent medial soft tissue because of superficial adhesions. (**d**), (**e**), and (**f**) Representative case of a good SSC sliding rate. (**d**) Internal rotation. (**e**) External rotation. (**f**) Sliding condition of the subscapularis tendon. During internal-to-external rotation, the SSC tendon slides independently with greater mobility. *SSC*, subscapularis.

### Statistical analysis

All statistical analyses were performed using EZR (Saitama Medical Center, Jichi Medical University, Saitama, Japan), which is a graphical user interface for R (The R Foundation for Statistical Computing, Vienna, Austria). More precisely, it is a modified version of R commander designed to add statistical functions frequently used in biostatistics.

Firth penalized logistic regression analysis was performed using sex, workers' compensation, presence of diabetes mellitus (DM), 1-month SSC sliding rate, and 1-month UER as independent variables.

To reduce potential bias between Group N and Group S and to evaluate diagnostic performance sensitivity, propensity score matching was employed. Propensity scores were estimated using a logistic regression model based on the following factors: age at surgery, sex, BMI, operative side, tear size, workers' compensation, and the presence of DM, which have been reported to be associated with POSS.^2^, ^3^, ^4^^,^^7^^,^^28^^,^^33^ Matching was performed in a one-to-one ratio with a caliper width of 0.2, and standardized mean differences were calculated to assess baseline covariate balance.

The Student *t*-test and Fisher exact test were performed to compare demographic data between the groups. The Student *t*-test was also employed to compare ER, UER, and SSC sliding rate between the groups. A post-hoc power analysis was performed based on SSC sliding rate, UER, and ER between the groups by use of an α value of 0.05. Cohen d effect sizes using the pooled standard deviations were calculated to assess the magnitude of differences in groups between each variable before and after propensity score matching. The strength of effect sizes was interpreted as small (d < 0.40), medium (0.40 ≤ d < 0.80), or large (d ≥ 0.80).^18^

For variables showing significant differences between groups after propensity score matching, receiver operating characteristic (ROC) curve analysis and the Youden J statistic were used to evaluate predictive thresholds for POSS of SSC sliding rate and UER and ER data before propensity score matching.

Inter-rater and intrarater reliabilities (intraclass correlation coefficients [ICCs]) were evaluated in 30 shoulders randomly selected from the overall study cohort by independent physical therapists who were not involved in the study and were blinded to patient identities. The assessments were independently conducted by 2 physical therapists who were blinded to each other's measurements and patient clinical information. To evaluate intrarater reliability, 1 therapist repeated the measurement at least seven days after the initial assessment, while remaining blinded to the first assessment results. To evaluate inter-rater reliability, a second examiner independently analyzed a subset of the recorded video clips after both examiners had standardized the criteria for identifying the point of sliding cessation. ICC (1.1) and ICC (2.1) were calculated for SSC sliding rate and UER. *P* values < 0.05 were considered statistically significant. Because ER, UER, and SSC sliding rate were assessed at 2 post-operative time points (1 and 3 months), Bonferroni correction was applied for these repeated comparisons when appropriate, with the original *P* values multiplied by 2.

## Results

In total, 125 shoulders that underwent ARCR between April 2017 and June 2018 were identified. Of these, 35 shoulders were excluded due to isolated SSC tendon repair (n = 6), massive/irreparable rotator cuff tear (n = 1), follow-up duration of less than 12 months (n = 13), and incomplete ROM data (n = 15) (Fig. 3).

**Figure 3:**
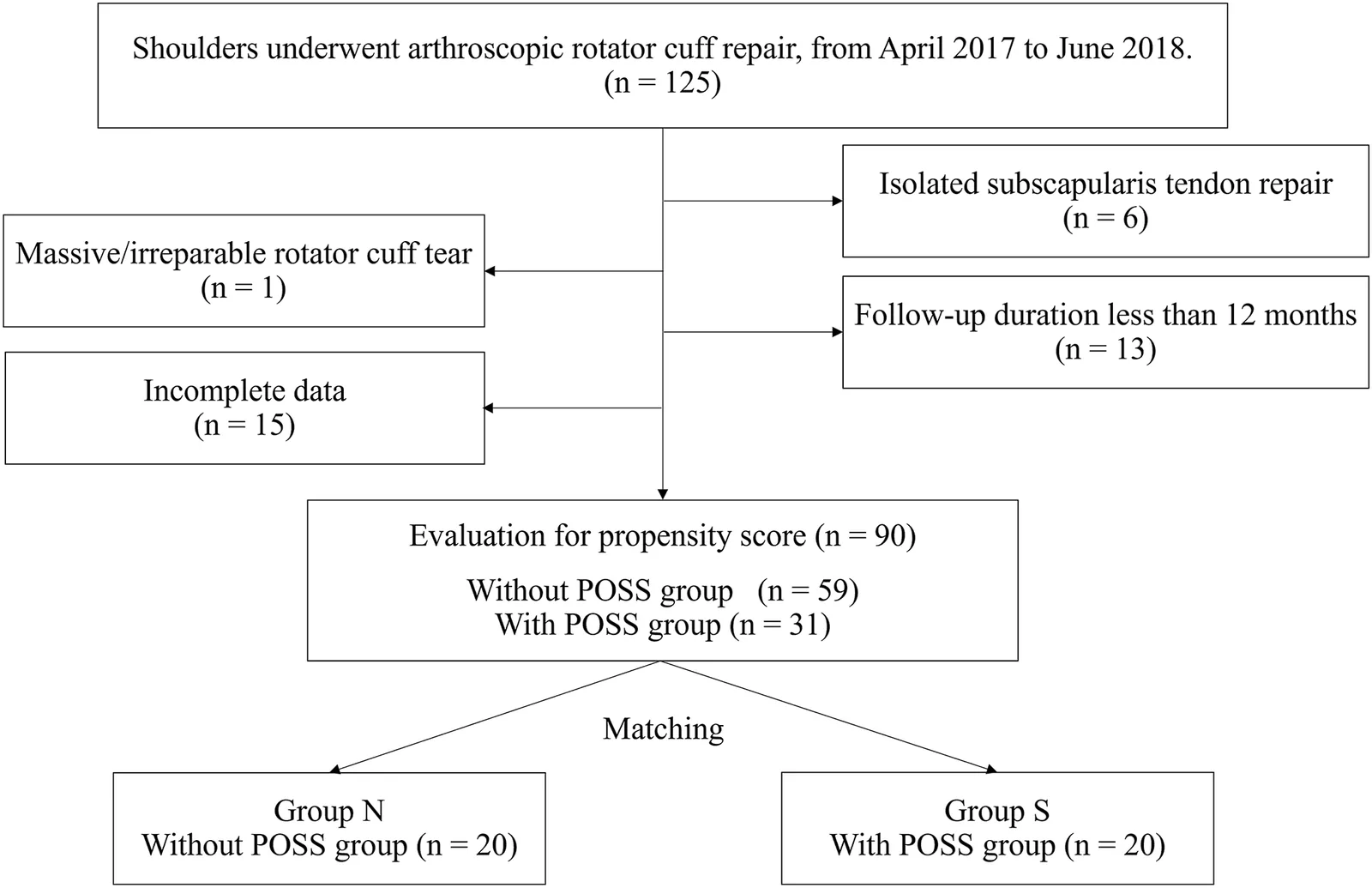
Flowchart of the patient selection. *SSC*, subscapularis; *POSS*, post-operative shoulder stiffness.

Among the remaining 90 shoulders, 59 were classified into Group N and 31 were classified into Group S. Regarding SSC tendon rupture, Group N and Group S included 19 and 12 patients before propensity score matching, and 8 and 10 patients after matching, respectively. In group S, 3 shoulders had flexion less than 120°, and all of them had ER less than 30°. Significant differences were observed in sex, the presence of diabetes, and workers' compensation, and all ROM before propensity score matching (Table I). After propensity score matching, 40 shoulders were included in the analysis, with 20 in Group N and 20 in Group S. The demographic and pre-operative clinical characteristics were similar between the groups (Table II).

**Table I:** Demographic data and post-operative range of motion prior to propensity score matching.

Variable	Group N n = 59	Group S n = 31	*P* value
Sex, male/female	32/27	25/6	**.021**
Age at surgery, yr	63 ± 11	65 ± 10	.319
BMI, kg/m^2^	24.0 ± 2.7	24.0 ± 3.3	.969
Operative side, right/left	42/17	17/14	.162
Diabetes mellitus, yes/no	4/55	7/24	**.043**
Workers' compensation, yes/no	0/59	4/27	**.012**
Rotator cuff tear size, partial/small/medium/large	9/19/22/9	4/6/9/12	.101
The presence of subscapularis tendon tear, yes/no	40/19	19/12	.642
CHL release, yes/no	47/12	24/7	.792
Capsule release, yes/no	50/9	24/7	.399
LHB management, absence of LHB/preserve/tenodesis/tenotomy	2/13/35/9	1/3/17/10	.182
Pre-operative passive range of motion			
Abduction, degree	123 ± 39	135 ± 31	.618
Forward elevation, degree	139 ± 29	135 ± 31	.484
External rotation, degree	46 ± 14	29 ± 15	**<.001**
Internal rotation, cm	30 ± 10	36 ± 13	**.013**
Post-operative passive range of motion at 12 mo after surgery			
Abduction, degree	156 ± 19	140 ± 23	**.001**
Forward elevation, degree	159 ± 14	148 ± 17	**.002**
External rotation, degree	50 ± 11	20 ± 7	**<.001**
Internal rotation, cm	27 ± 8	32 ± 9	**.009**

*BMI*, body mass index; *CHL*, coracohumeral ligament; *LHB*, long head of the biceps.

The values are given as mean ± standard deviation.

Bold text indicates a significant *P* value.

**Table II:** Demographic data and post-operative range of motion of the matched patients.

Variable	Group N n = 20	Group S n = 20	*P* value	SMDs
Sex, male/female	17/3	14/6	1.000	0.107
Age at surgery, yr	64 ± 11	65 ± 11	.648	0.145
BMI, kg/m^2^	24.3 ± 2.7	23.8 ± 3.4	.575	0.179
Operative side, right/left	14/6	13/7	1.000	0.107
Diabetes mellitus, yes/no	3/17	2/18	1.000	0.152
Workers' compensation, yes/no	0/20	0/20	1.000	<0.001
Rotator cuff tear size, Partial/small/medium/large	2/3/11/4	4/3/6/7	.462	0.565
The presence of subscapularis tendon tear, yes/no	12/8	10/10	.751	
CHL release, yes/no	15/5	14/6	1.000	
Capsule release, yes/no	15/5	15/5	1.000	
LHB management, absence/preserve/tenodesis/tenotomy	1/1/15/3	0/2/9/9	.078	
Pre-operative passive range of motion				
Abduction, degree	125 ± 43	127 ± 36	.906	
Forward elevation, degree	139 ± 33	137 ± 31	.858	
External rotation, degree	44 ± 12	28 ± 16	**.001**	
Internal rotation, cm	30 ± 10	33 ± 14	.467	
Post-operative passive range of motion at 12 mo after surgery				
Abduction, degree	154 ± 20	141 ± 24	.068	
Forward elevation, degree	163 ± 10	149 ± 19	**.006**	
External rotation, degree	43 ± 10	20 ± 6	**<.001**	
Internal rotation, cm	26 ± 9	29 ± 9	.226	

*BMI*, body mass index; *CHL*, coracohumeral ligament; *LHB*, long head of the biceps; *SMDs*, standardized mean differences.

The values are given as mean ± standard deviation.

Bold text indicates a significant *P* value.

In the Firth penalized logistic regression analysis, sex (odds ratio: 5.69, *P* = .006), presence of DM (odds ratio: 0.21, *P* = .045), and 1-month SSC sliding rate (odds ratio: 1.04, *P* = .001) were identified as significant independent variables (Table III).

**Table III:** Firth penalized logistic analysis for predicting the POSS.

Variable	Odds ratio (95% CI)	*P* value
Sex	5.69 (1.58-27.1)	**.006**
Presence of diabetes mellitus	0.21 (0.04-0.97)	**.045**
Workers' compensation	0.07 (0.01-1.26)	.074
SSC sliding rate at 1 mo	1.04 (1.01-1.07)	**.001**
External rotation with ultrasonography at 1 mo	1.03 (0.99-1.07)	.076

*SSC*, subscapularis; *CI*, confidence interval; *POSS*, post-operative shoulder stiffness.

Bold text indicates a significant *P* value.

Regarding the SSC sliding rate, UER, and ER, all variables at post-operative 1 and 3 months were significantly greater in Group N than in Group S before propensity score matching (Table IV). After propensity score matching, the SSC sliding rate at post-operative 1 and 3 months, UER at post-operative 3 months, and ER at post-operative 1 and 3 months were significantly greater in Group N than in Group S (66 ± 21% vs. 35 ± 31%, *P* = .001; 70 ± 24% vs. 43 ± 32%, *P* = .009; 10 ± 10° vs. −5 ± 21°, *P* = .012; 13 ± 12° vs. −1 ± 17°, *P* = .013; and 25 ± 10° vs. 16 ± 12°, *P* = .022, respectively) (Table V). Post hoc power analysis demonstrated a power of 0.904 for the SSC sliding rate at post-operative 1 month (Table V). With respect to Cohen d effect sizes, almost all variables demonstrated large effects. The largest effect sizes were observed for UER at post-operative 3 months before propensity score matching and for the SSC sliding rate at post-operative 1 month after propensity score matching.

**Table IV:** SSC sliding rate, external rotation with ultrasonography, and external rotation at the side prior to propensity score matching.

Variable	Group N n = 59	Group S n = 31	*P* value	Effect size (Cohen d)
One month after surgery				
SSC sliding rate, %	63 ± 26	37 ± 31	**<.001**	1.07 (0.60-1.54)
External rotation with ultrasonography, degree	6 ± 17	−10 ± 20	**<.001**	0.86 (0.40-1.31)
External rotation at the side, degree	18 ± 17	1 ± 17	**<.001**	0.99 (0.52-1.45)
Three months after surgery				
SSC sliding rate, %	63 ± 25	46 ± 33	**.004**	0.71 (0.25-1.16)
External rotation with ultrasonography, degree	19 ± 13	−3 ± 18	**<.001**	1.52 (1.02-2.01)
External rotation at the side, degree	33 ± 16	15 ± 12	**<.001**	1.24 (0.77-1.72)

*SSC*, subscapularis.

The values are given as mean ± standard deviation.

Bold text indicates a significant *P* value.

*P* values were adjusted using Bonferroni correction for 2 comparisons.

**Table V:** SSC sliding rate, external rotation with ultrasonography, and external rotation at the side of the matched patients.

Variable	Group N n = 20	Group S n = 20	*P* value	Effect size (Cohen d)	Power
One month after surgery					
SSC sliding rate, %	66 ± 21	35 ± 31	**.001**	1.19 (0.49-1.88)	0.904
External rotation with ultrasonography, degree	−1 ± 15	−10 ± 20	.204	0.53 (0.12-1.18)	0.360
External rotation at the side, degree	13 ± 12	−1 ± 17	**.013**	0.91 (0.24-1.58)	0.781
Three months after surgery					
SSC sliding rate, %	70 ± 24	43 ± 32	**.009**	0.96 (0.28-1.64)	0.789
External rotation with ultrasonography, degree	10 ± 10	−5 ± 21	**.012**	0.92 (0.25-1.59)	0.781
External rotation at the side, degree	25 ± 10	16 ± 12	**.022**	0.85 (0.18-1.52)	0.651

The values are given as mean ± standard deviation.

Bold text indicates a significant *P* value.

*P* values were adjusted using Bonferroni correction for 2 comparisons.

*SSC*, subscapularis.

According to ROC curve analysis, the thresholds for POSS 1 year post-operatively were 48% for the SSC sliding rate and 6° for ER at 1 month after surgery. At 3 months post-operatively, the thresholds were 38% for SSC sliding rate, 7° for UER, and 17° for ER (Table VI) (Fig. 4).

**Table VI:** Predictive threshold values for predictors identified from ROC curve analysis.

Variable	Threshold values	AUC (95% CI)	Sensitivity, % (95% CI)	Specificity, % (95% CI)	PPV, % (95% CI)	NPV, % (95% CI)
One month after surgery						
SSC sliding rate, %	48	0.74 (0.62-0.86)	65 (45-81)	83 (71-92)	67 (47-83)	82 (70-91)
External rotation at the side, degree	6	0.75 (0.65-0.86)	65 (45-81)	78 (65-88)	61 (42-77)	81 (68-90)
Three months after surgery						
SSC sliding rate, %	38	0.67 (0.53-0.80)	52 (33-70)	95 (86-99)	84 (60-97)	79 (68-88)
External rotation with ultrasonography, degree	7	0.85 (0.75-0.94)	84 (66-95)	86 (75-94)	77 (59-89)	91 (80-97)
External rotation at the side, degree	17	0.83 (0.73-0.93)	77 (61-92)	86 (75-94)	75 (57-89)	88 (77-95)

*SSC*, subscapularis; *AUC*, area under the curve; *ROC*, receiver operating characteristic; *CI*, confidence interval; *PPV*, positive predictive value; *NPV*, negative predictive value.

**Figure 4:**
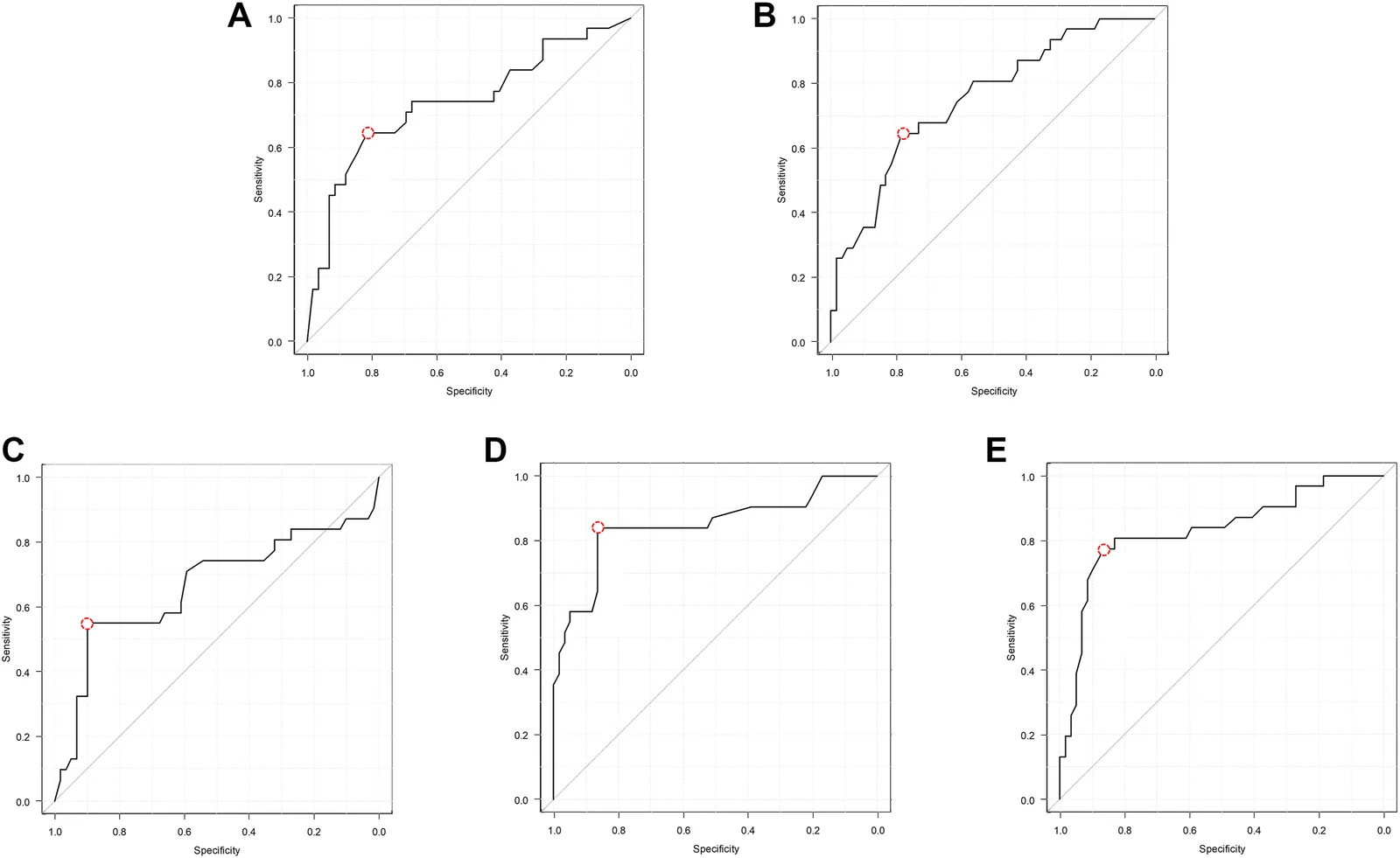
ROC curve. (**a**) SSC sliding rate at 1 month post-operatively. (**b**) ER at 1 month post-operatively. (**c**) SSC sliding rate at 3 months post-operatively. (**d**) UER at 3 months post-operatively. (**e**) ER at 3 months post-operatively. *SSC*, subscapularis; *ROC*, receiver operating characteristic; *UER*, ultrasonographic external rotation; *ER*, external rotation.

ICC (1.1) and ICC (2.1) for SSC sliding rate were 0.87 (95% confidence interval [CI]: 0.75-0.94) and 0.72 (95% CI: 0.45-0.86), respectively. ICC (1.1) and ICC (2.1) for UER were 0.97 (95% CI: 0.92-0.99) and 0.97 (95% CI: 0.92-0.99), respectively. According to the criteria of Landis classification, it ranged from “substantial” to “almost perfect.”

## Discussion

This study demonstrated that ultrasonographic evaluation is effective in predicting the occurrence of POSS at 1 year after surgery, and that this newly developed ultrasonographic measurement method showed excellent intrarater and inter-rater reliability. Reduced SSC sliding rate and limited UER in the early post-operative period were associated with POSS at 12 months post-operatively.

Because POSS adversely affects outcomes and may necessitate arthroscopic capsular release, identifying its risk factors is crucial.^1^^,^^3^^,^^4^^,^^15^^,^^23^ Although age, sex, cuff tear size, rehabilitation, and presence of diabetes have been implicated, the exact causes remain unclear because of the multifactorial etiology of POSS.^2^, ^3^, ^4^^,^^7^^,^^28^^,^^33^ Before propensity score matching, significant intergroup differences were observed in sex, diabetes, and workers' compensation, consistent with previous reports.

Involvement of anterosuperior soft-tissue structures has been reported as a cause of POSS.^5^^,^^8^^,^^9^^,^^13^^,^^22^^,^^30^ However, objective *in vivo* methods linking soft-tissue stiffness to POSS have been lacking. Our findings show that shoulders with POSS exhibited significantly reduced SSC sliding rates as early as 1 month post-operatively, indicating a strong early association with the development of POSS.

The greatest difference in SSC sliding rate occurred at 1 month post-operatively, whereas the largest difference in UER was identified at 3 months. This temporal shift aligns with the biological healing process, as repaired tendon maturation and peritendinous adhesions progress over approximately 7 weeks.^10^ In the early phase, when adhesions are still forming, SSC sliding rate may reflect subtle soft-tissue restrictions more sensitively than ER or UER. By 3 months, as stiffness becomes more established, UER—which isolates glenohumeral motion—may more accurately capture these structural restrictions.

Diagnostic thresholds further supported these temporal differences. At 1 month, SSC sliding rate and ER demonstrated comparable AUC values; however, SSC sliding rate showed higher specificity, suggesting that it may be preferable for early risk stratification. At 3 months, UER demonstrated superior overall predictive performance in terms of AUC, sensitivity, and specificity. Thus, early assessment of SSC sliding rate at 1 month may be particularly valuable for identifying high-risk patients and guiding timely interventions.

Regarding measurement reliability, intrarater reliability was classified as "almost perfect" and inter-rater reliability as "substantial" based on Landis criteria.^14^ Similar reliability for soft-tissue sliding has been reported in other anatomical regions,^26^^,^^27^ indicating that ultrasonography assessment of SSC sliding is sufficiently reliable for clinical application.

This study has several limitations. First, it was conducted at a single center, which may limit generalizability. Second, despite propensity score matching, residual confounding cannot be completely excluded. Third, we did not evaluate pre-operative stiffness, psychological factors, or pain-related behaviors. Fourth, because workers' compensation exhibited complete separation and sex exhibited quasi-complete separation, we performed Firth penalized logistic regression analysis to address these analytical limitations. However, the model produced unstable estimates, such as DM unexpectedly appearing to reduce the risk of POSS, which contradicts previous literature. Although propensity score matching has inherent limitations in the setting of a small sample size, it allowed us to account for a broader set of potential confounders without including numerous covariates directly in the outcome model. Therefore, we based our primary conclusions on the propensity score–matched analysis. Fifth, because of the small sample size, pre-operative ER ROM could not be adjusted via propensity score matching, warranting future larger studies. Sixth, the standardized mean difference for tear size remained relatively high after matching. Seventh, after matching, the sample size was reduced to 40 shoulders (20 per group). Although statistically significant differences were detected, estimates derived from such a small cohort—including ROC-derived thresholds, reliability, and effect sizes—may be unstable. Furthermore, these thresholds lack external validation. Therefore, these proposed cutoff values must be considered preliminary rather than definitive, and future multicenter validation studies are required before recommending their routine clinical use. Finally, whether implementing targeted early interventions based on these thresholds improves outcomes remains unproven, requiring future prospective trials.

## Conclusion

This study demonstrated that early post-operative ultrasonographic evaluation is useful for predicting the development of POSS at 1 year after ARCR. Reduced SSC sliding rate and limited UER in the early post-operative period were associated with POSS at 12 months after ARCR. This novel ultrasonographic assessment demonstrated high reliability and may be a valuable tool for early risk stratification and post-operative management.

## Disclaimers:

Funding: No funding was disclosed by the authors.

Conflicts of interest: Norimasa Takahashi has received speaker honoraria from DePuy Synthes and Medacta international outside the submitted work. Hiroyuki Sugaya has received speaker honoraria from DePuy Synthes, Smith & Nephew, Zimmer, Biomet, and Stryker outside the submitted work. Any additional authors, their immediate families, and any research foundations with which they are affiliated have not received any financial payments or other benefits from any commercial entity related to the subject of this article.
